# A possible connection between psychosomatic symptoms and daily rhythmicity in growth hormone secretion in healthy Japanese students

**DOI:** 10.1186/1740-3391-7-10

**Published:** 2009-08-05

**Authors:** Mitsuo Nagane, Kazunori Yoshimura, Shu-Ichi Watanabe, Masahiko Nomura

**Affiliations:** 1 Department of Educational Physiology, Chiba University, Chiba 263-8522, Japan; 2 Department of Rehabilitation, Nihon Institute of Medical Science, Japan; 3 Department of Physiology, Saitama Medical University, Japan; 4 International Education and Training Center, Saitama Medical University, Japan

## Abstract

**Background:**

Students suffering from psychosomatic symptoms, including drowsiness and feelings of melancholy, often have basic lifestyle problems. The aim of this study was to investigate whether psychosomatic complaints may be related to circadian dysfunction.

**Methods:**

We examined 15 healthy students (4 men and 11 women) between 21 and 22 years old. To assess the presence of psychosomatic symptoms among the subjects, we developed a self-assessment psychosomatic complaints questionnaire consisting of five items pertaining to physical symptoms and five items concerning mental symptoms. The subjects rated their psychosomatic symptoms twice a day (08:00 and 20:00 h). We also assessed growth hormone secretion patterns by fluorescence enzyme immunoassay (FEIA). Salivary samples were collected from the subjects at home five times a day (20:00, 24:00, 04:00, 08:00, and 12:00 h) in Salivette tubes.

**Results:**

The results indicated a relationship between the self-assessment scores and the salivary levels of growth hormone. Subjects with high self-assessment scores showed significant variability in growth hormone secretion over the day, whereas subjects with low self-assessment scores did not.

**Conclusion:**

Psychosomatic symptoms may be associated with circadian dysfunction, as inferred from blunted rhythmicity in growth hormone secretion.

## Background

Japanese students suffering from psychosomatic disorders, such as those involving mood and sleep, may exhibit basic problems in their lifestyle, including deleterious changes in their living environment and dietary or lifestyle disturbances [1]. In particular, staying up late is associated with decreased appetite and missed breakfast the following morning, irregular bowel movements and sleepiness. Perhaps the biggest problem facing today's Japanese students is their lack of daily physical exercise, brought on by stressful academic courses over long periods of time, too much television and computer games and increased automobile use [2]. Many Japanese youngsters stay up late at night [3].

A circadian pacemaker in the central nervous system regulates human sleep cycles, hormone secretion, subject alertness, objective performance levels and other physiologic functions over a 24-h period. Core body temperature, plasma cortisol, and plasma melatonin are three variables frequently used to estimate the phase of the human pacemaker [4], although many other hormones, including growth hormone, exhibit daily rhythmicity. Technical advances that make the assessment of biomarkers in saliva possible have enabled researchers to non-invasively study biosocial processes related to stress in naturalistic contexts. Chiappin et al [5] showed the usefulness and possibility of salivary hormone analysis containing growth hormone. Rantonen [6] found a linear correlation between salivary and serum growth hormone.

Carroll et al. [7] described negative effects of growth hormone insufficiency on psychological well-being, including reduced vitality and energy, depressed mood, emotional lability, impaired self-control, anxiety, and increased social isolation. Patients with growth hormone deficiencies report decreased energy levels, greater emotional lability, increased difficulties with sexual relationships and a greater sense of social isolation than control subjects [8]. However, no direct relationship has been shown between growth hormone deficiency and psychometrically measured depression, apathy or psychosomatic well-being [9].

The purpose of the present study was to investigate individual variation in the levels of growth hormone in healthy subjects and to examine the relationship between an individual's hormone profile and his or her psychosomatic complaints.

## Methods

### The subjects and self-assessment questionnaire

Fifteen subjects (4 men and 11 women) without major medical disorders ranging in age from 21 to 22 years participated in this study. The study design was approved by the Ethics Committee of Chiba University, Japan, and informed consent was obtained from all subjects. A self-assessment questionnaire concerning psychosomatic symptoms was developed in accordance with data from the Health Behavior in School-Aged Children (HBSC) study of the WHO [10]. The questionnaire for this study contained five items related to physical symptoms and five items pertaining to mental symptoms (Table 1). The questionnaire was used to measure each individual's psychosomatic symptoms at home twice each day (08:00 and 20:00 h). The items were rated on a 4-point scale, with 1 = not true at all and 4 = completely true. The total score for the 10-item scale ranged from 10 to 40, with higher scores indicating a greater degree of psychosomatic complaints. The subjects were allocated post hoc (median split) to a High (n = 7) or Low (n = 8) Self-Assessment Group based on their total morning score (with higher scores corresponding to lower self-assessment).

**Table 1:** Morning and evening psychosomatic condition scores collected from the self-assessment psychosomatic complaint questionnaire

	High Self-Assessment Group (n = 7)		Low Self-Assessment Group (n = 8)		Morning Comparison
	
	**Morning** **(08:00 h)**	Evening (20:00 h)	**Morning** **(08:00 h)**	Evening (20:00 h)	***t*-value**
• Physical symptoms					
1. Drowsiness	**3.14**	1.43	**3.38**	1.50	**-0.67**
2. Poor appetite	**2.14**	1.86	**2.25**	1.38	**-0.30**
3. Heaviness in the head	**1.14**	1.29	**1.38**	1.13	**-1.00**
4. Dizziness	**1.00**	1.00	**1.75**	1.25	-3.00*
5. Whole-body fatigue	**2.00**	1.57	**2.63**	1.75	**-1.49**
					
• Mental symptoms					
6. Lack of motivation	**2.00**	2.00	**2.75**	2.13	-1.82
7. Easily irritated	**1.00**	1.14	**1.50**	1.50	-2.65*
8. Feelings of melancholy	**1.00**	1.14	**1.88**	2.13	-2.97*
9. Desire to rest	**2.00**	2.00	**3.00**	2.50	-2.45*
10. Anxiety	**1.14**	1.57	**2.50**	3.00	-3.80**

total	**19.00**	16.60	**32.75**	23.00	-3.96**

The scores shown are means. Each scale ranges from 1 to 4, with higher scores indicating a greater degree of psychosomatic symptoms (No, Somewhat No, Somewhat Yes, Yes).

**p < 0.01; *p < 0.05

### Sample collection

Saliva was collected into Salivette tubes (Sarstedt, Germany) using polyester swabs from the subjects' mouths following 2 min of chewing. Samples were collected five times a day at home (20:00, 24:00, 04:00, 08:00, and 12:00 h). Both the day of sampling and the preceding day were required to be normal days (i.e., without special events or stressful circumstances). After sample collection, the saliva was stored at -20°C until being analysed.

### Salivary growth hormone assay

On the day of testing, the samples were centrifuged at 3,000 rpm for 10 min to remove all mucin. A standard fluorescent determination immunoassay was used to assess the growth hormone concentrations in each sample. To avoid inter-assay variability, all determinations were performed in a single series. In the first step, 96-well fluoro-nunc plates (Nunc, Black MicroWell 137101, Denmark) were pre-coated with 100 μl of anti-growth hormone antibody (Quartett, 2071800210, Germany) and incubated for 1.5 h at room temperature. After incubation, the plate was washed three times with phosphate-buffered saline and blocked for 1 h. After washing, 100 μl of saliva or a standard solution was dispensed into each well and left for 1.5 h. After washing, primary antibody (Funakoshi, FU47500254, Japan) was added to the plates and incubated for 1.5 h. Next, incubation with a secondary antibody (Novus Biologicals, NB120-7112, USA) was performed for 1 h. After washing, rabbit anti-ovine immunoglobulin (Amersham Biosciences, ECF Western Blotting Reagent Pack, USA) was added. After 20 min of incubation, the plate was scanned using a Fluoromark Microplate Fluorometer (Bio-Rad, USA) with excitation at 485 nm and emission at 590 nm.

### Data analysis

The significance of differences between group means was tested by analysis of variance (ANOVA), followed by protected *t *tests when appropriate. The presence of daily rhythmicity in salivary growth hormone was tested by ANOVA and by the cosinor procedure [11].

## Results

### Self-assessment psychosomatic complaints questionnaire

A factorial repeated measures ANOVA (high/low self-assessment versus morning/evening self-assessment scores) was conducted. ANOVA results showed significant differences (p < .05) between morning and evening self-assessment scores. Post hoc analyses revealed that, as shown in Table 1, the high self-assessment group (total 19.00) differed significantly from the low self-assessment group (total 32.75) in terms of their morning scores (Welch's *t*-test, *t *= -3.96, df = 7.57, p < 0.01). The low self-assessment group subjects complained of negative psychosomatic conditions including being easily irritated (p < .05), feeling melancholy (p < .05), having a desire to rest (p < .05), and feeling anxious (p < .01).

### Assessment of daily rhythmicity of salivary growth hormone secretion

**Figure 1:**
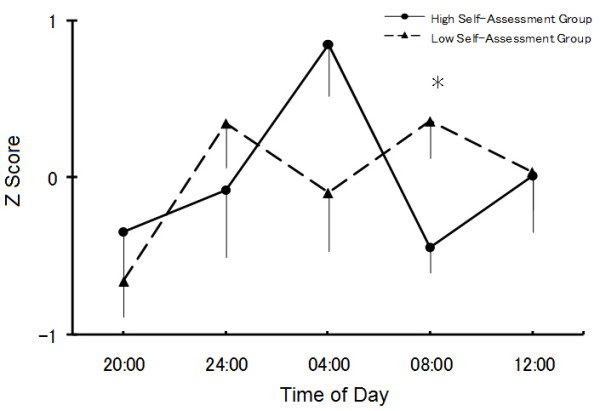
**Daily variation in salivary level of growth hormone in the high and low self-assessment groups**. The results are presented as means ± S.E.M. * p < .05.

We collected saliva profiles from 15 healthy students (4 men and 11 women). The amplitude of salivary growth hormone, defined as the difference between the highest and lowest salivary concentrations, was used to produce a standardisation, or Z, score. As shown in Figure 1, noticeable variation was observed in the hormonal rhythms of the subjects, including differences in the salivary growth hormone secretion profiles of the high and low self-assessment groups. Cosinor analysis revealed no significant 24-hour rhythmicity in the secretion profiles of either group (p > .50), but a repeated measures ANOVA identified statistically significant (p < .05) time-related variations for growth hormone in the high self-assessment group. The secretion profile of the low self-assessment group did not exhibit the typical, sharp peak in the early morning [12], and ANOVA showed no time-related variation (p > .10). At 08:00 h, salivary growth hormone levels were significantly lower (p < .05) in the low self-assessment group than in the high self-assessment group.

## Discussion

Psychosocial factors have been previously shown to affect the psychosomatic symptoms reported by Japanese school children [13]. Psychosomatic symptoms, which are largely mediated by the autonomic nervous system, are strongly influenced by an individual's lifestyle, and the current so-called 24-h society in Japan may have changed the environmental conditions of students. More than 80% of school refusal cases (school phobia) suffer from sleep disorders, with a tendency towards day/night reversal and easy fatigability, especially during the period immediately following their school social life [14]. Thus, impairment in circadian rhythmicity may be a cause of school refusal in Japan. The present study was the first step in an attempt to investigate this hypothesis.

Our assessment of salivary growth hormone secretion was not sensitive enough to detect significant daily rhythmicity, but the highest level measured in the subjects of our high self-assessment group occurred earlier in the day than the peak of the daily rhythm measured in a previous study [15]. Peak hormonal secretions often shift to the morning if an activity continues long into the night. A link between deficiency of growth hormone and reduced quality of life or well-being has been reported by many researchers [7].

Our results indicate a relationship between the self-assessment scores and the levels of growth hormone. Subjects with high self-assessment scores in the morning showed significant variability in growth hormone secretion during the day, whereas subjects with low self-assessment scores did not. Thus, psychosomatic symptoms may be associated with hormonal rhythms related to basic lifestyle habits.

Nocturnal melatonin secretion can be suppressed by exposure to light on the order of several hundred lux, such as ordinary room light [16]. Thus, a subject's pattern of melatonin secretion may reflect his or her life rhythm, and melatonin secretion appears to be an important index of circadian rhythmicity. Based on our previous finding that growth hormone and melatonin exhibit similar daily rhythmicity [17], we believe that estimates of the state of the central circadian clock can be most accurate if they are based on the analysis of the secretion patterns of both melatonin and growth hormone.

Some limitations of our study must be emphasized. First, it is possible that the sleep-disrupting effect of waking at 00.00 and 04.00 to produce a saliva sample had a disruptive effect on hormonal secretion. Second, we observed a larger difference in terms of gender than has been previously described [18], with women having sevenfold higher serum growth hormone concentrations than men during the day. Though we did not directly examine sex differences in growth hormone secretion, we recognise it as an important topic for further research.

## Conclusion

Psychosomatic symptoms may be associated with circadian dysfunction, as inferred from blunted rhythmicity in growth hormone secretion.

## Competing interests

The authors declare that they have no competing interests.

## Authors' contributions

MN designed the experiments, collected data and wrote the manuscript. KY managed the laboratory and adjusted the schedule of subjects. SW participated in the design of the study and performed statistical analysis. MN supervised the study. All authors read and approved the final version of the article.
